# Supplementary material for: Supraphysiological levels of GDF11 induce striated muscle atrophy
Source: EMBO Mol Med. 2017 Mar 7;9(4):531–44. doi: 10.15252/emmm.201607231 (PMC5376753; doi:10.15252/emmm.201607231)
Supplement: Supplementary file 1 — Expanded View Figures PDF [file EMMM-9-531-s001.pdf]

Expanded View Figures

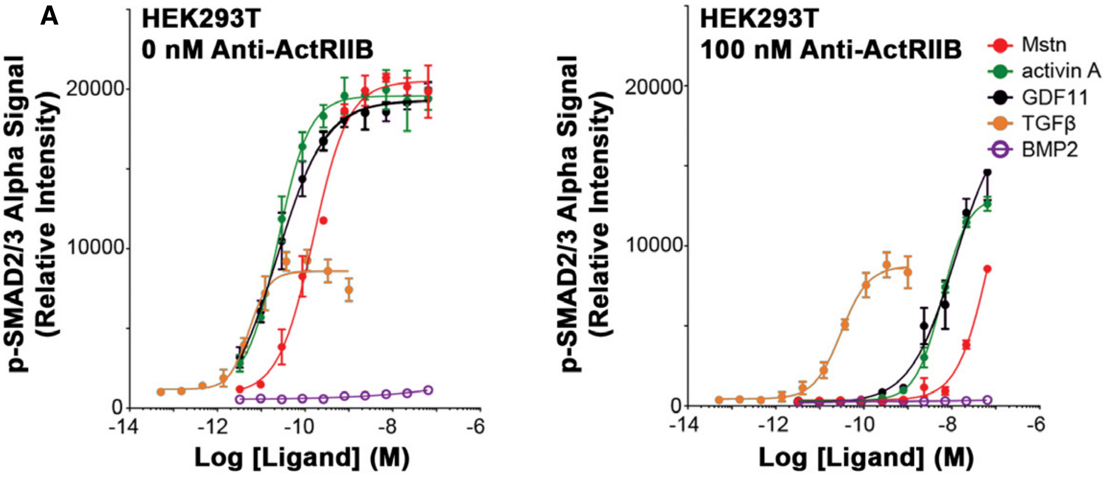

B

EC50, nM pSMAD2/3	0nM Anti-ActIIR	100nM Anti-ActIIR
Mstn	0.156 ± 0.017	72 ± 37
GDF11	0.023 ± 0.006	12 ± 3.8
TGFβ	0.005 ± 0.0006	0.030 ± 0.003
activinA	0.024 ± 0.003	6 ± 0.22

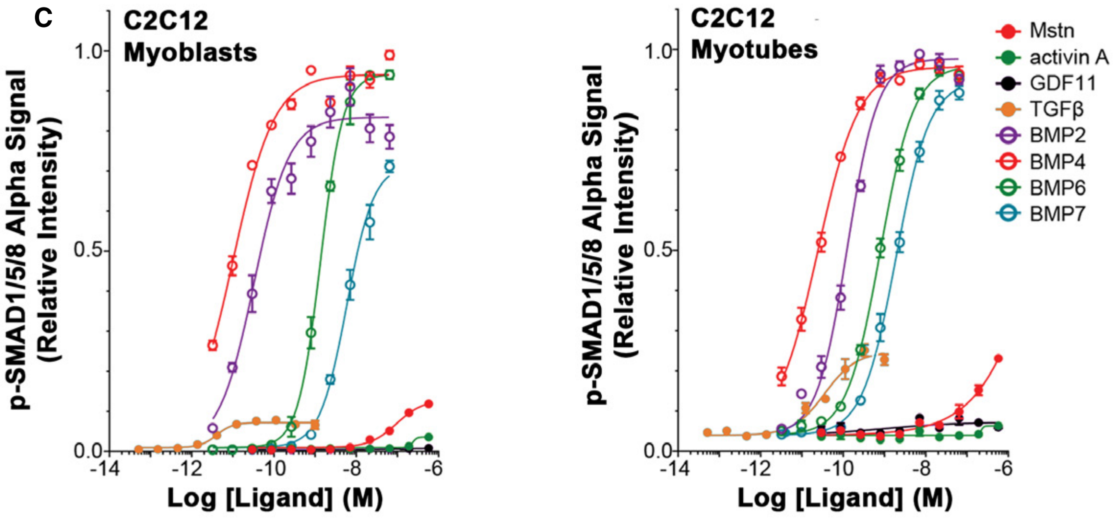

Figure EV1. Additional SMAD response assays to TGFβ ligands.

A, B Significant shifts in the response of HEK293T cells to Mstn, GDF11, and activin A are observed with the addition of ActRIIB antibody (anti-ActRIIB), when 0 or 100 nM anti-ActRIIB was applied to HEK293T cells overnight prior to ligand addition and AlphaLISA evaluation. Note that the 0 nM antibody treatment panel is the un-normalized form of Fig 1A left. EC_{50} values (in nM) are listed for the antibody data in (B).

C p-SMAD1/5/8 response of C2C12 myoblasts (left) and myotubes (right) in response to TGFβ family member ligands. All cells were stimulated with ligand 1 h prior to lysis and evaluation by AlphaLISA.

Data Information: Values are displayed as mean ± SEM; $n = 4$ for all data points.

© 2017 The Authors

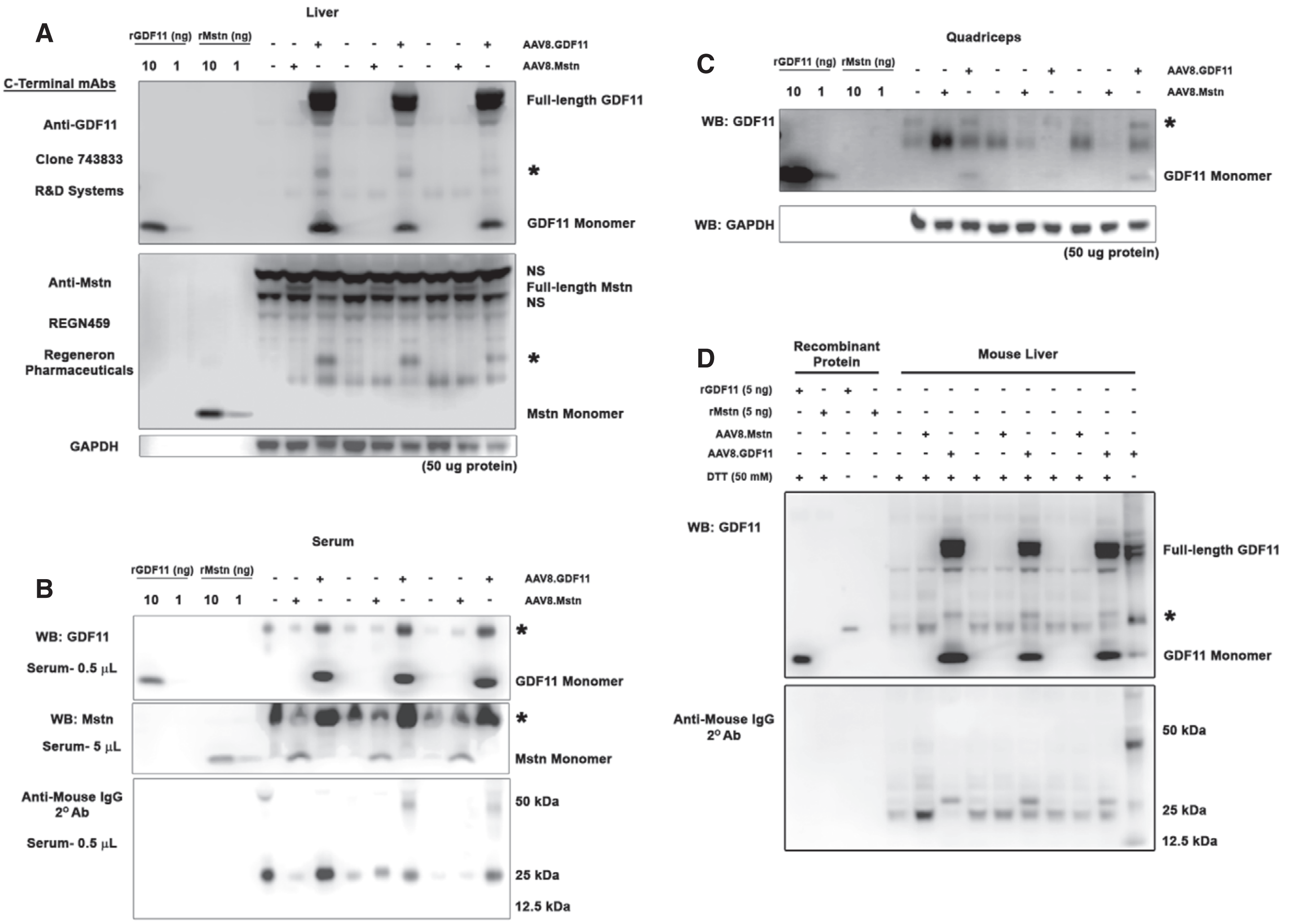

Figure EV2. Antibody validation and overexpression verification.

A–C Twelve-week-old C57BL/6 male mice were injected i.p. with PBS, a liver-specific myostatin (Mstn) construct packaged into AAV2/8 (AAV8.Mstn), or a liver-specific GDF11 construct packaged into AAV2/8 (AAV8.GDF11; n = 3). Verification of GDF11 and Mstn overexpression in the liver (A), serum (B), and quadriceps (C) of treated mice using clone 743833 anti-GDF11 (R&D Systems #MAB19581) and REGN459 anti-Mstn (Regeneron Pharmaceuticals) mouse monoclonal antibodies using samples pre-incubated with protein A/G-coated agarose beads, to reduce endogenous IgGs, and prepared in reducing conditions. The star (*) represents a 25 kDa band that is specifically prominent in AAV8.GDF11-treated samples, however, is detected by anti-mouse IgG secondary antibodies. Note that these are the full immunoblot images for those found in Fig 3B–D.

D Immunoblotting for GDF11 (using R&D #MAB19581) with reduced (50 mM DTT) and non-reduced (no DTT) forms of recombinant GDF11 and Mstn, and liver samples from control, AAV8.Mstn, and AAV8.GDF11 mice subjected to IgG depletion with both protein A/G (targets IgG heavy chains) and protein L (targets IgG light chains)-coated agarose beads. The differential detection of anti-mouse IgG immunoreactive bands between AAV8.GDF11-treated samples and the other groups indicates that GDF11 modifies anti-mouse IgG immunoreactive species which do not appear to be depletable by incubation with protein A/G or L. The non-reduced AAV8.GDF11 sample (lane 14) demonstrates both 25 and 12.5 kDa bands immunoreactive with anti-mouse IgG secondary antibodies, as well. The star (*) represents the ambiguous 25 kDa band mentioned above.

 © 2017 The Authors

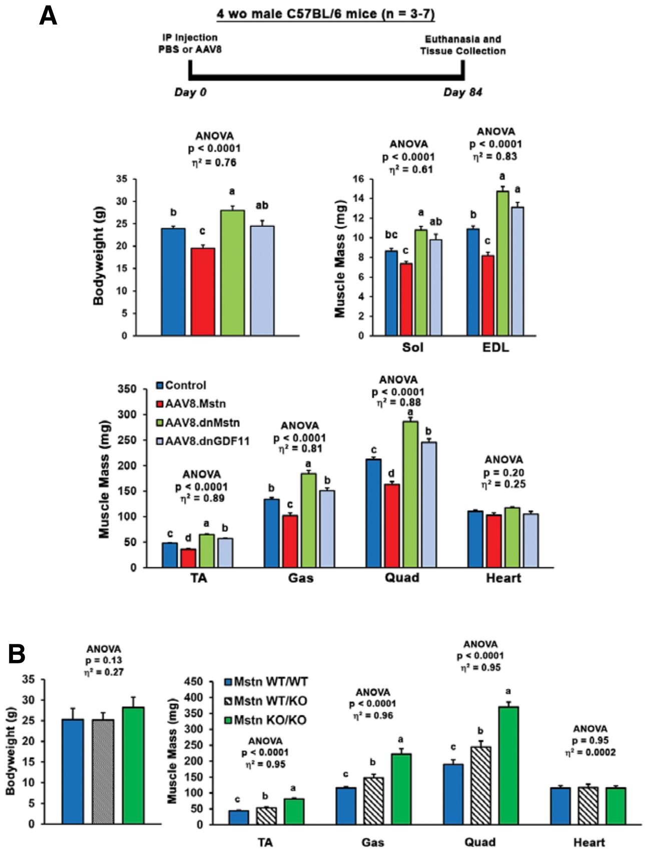

Figure EV3. Long-term Mstn/GDF11 manipulation *in vivo*.

A Four-week-old C57BL/6 male mice were injected IP with PBS (control; *n* = 6) or 1 × 10^{12} gc of AAV2/8 packaged liver-specific constructs of full-length Mstn (AAV8.Mstn; *n* = 7), Mstn D76A propeptide (AAV8.dnMstn; *n* = 3), or GDF11 D120A propeptide (AAV8.dnGDF11; *n* = 4) and euthanized at 16 weeks of age (84 day treatments).

B Body weights and muscle masses of 12-week-old $Mstn^{WT/WT}$ (*n* = 6), $Mstn^{WT/KO}$ (*n* = 6), and $Mstn^{KO/KO}$ (*n* = 4) mice congenic on a C57BL/6 background.

Data Information: Values are presented as mean ± SEM. Statistical analysis performed using one-way ANOVA analysis with Tukey's HSD *post hoc* test (non-connecting letters indicate $P < 0.05$ between groups) and effect size presented as eta-squared (η^2).

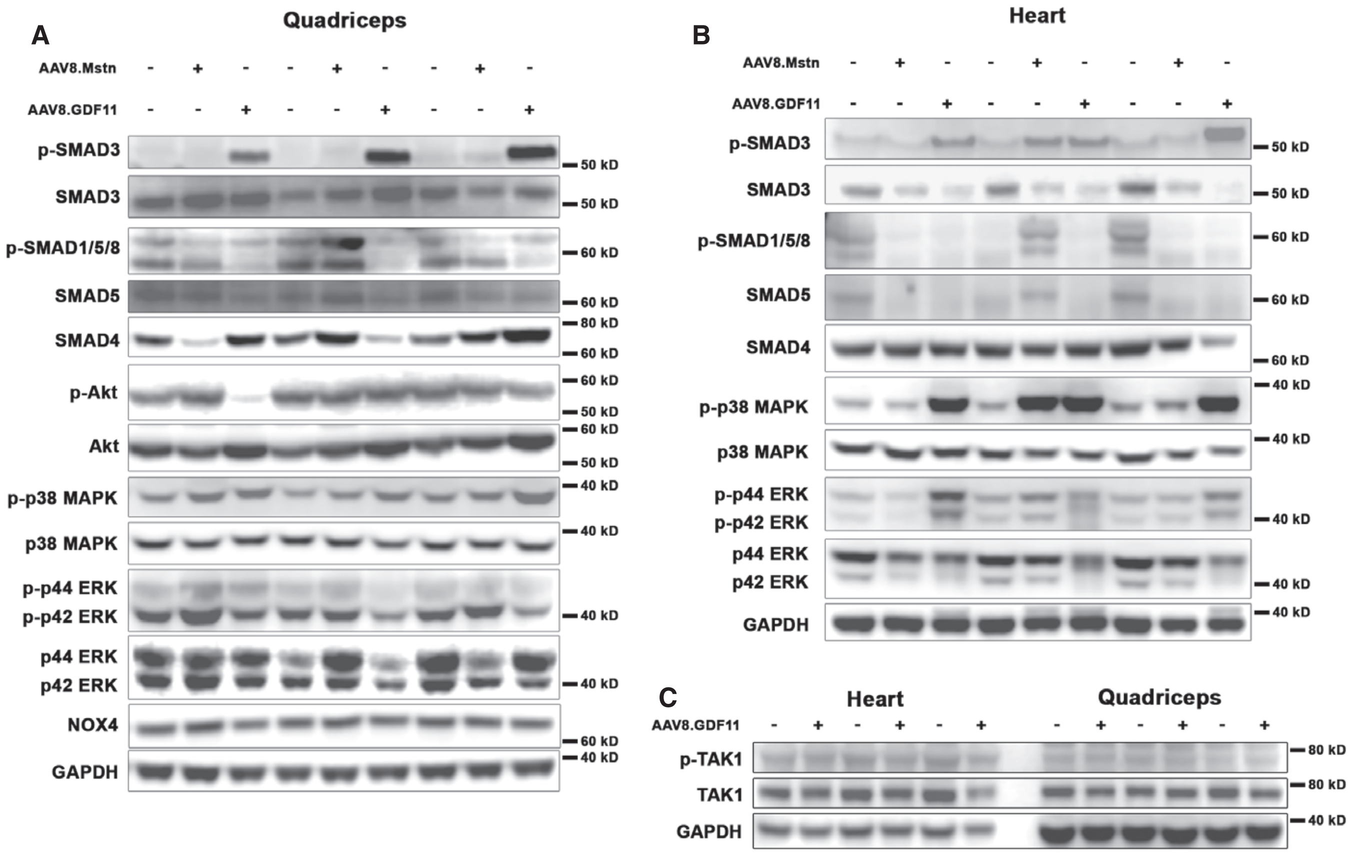

Figure EV4. Full immunoblots from AAV8.Mstn- and AAV8.GDF11-treated mice.

A, B Quadriceps (A) and heart (B) lysates from mice described in Figs 2 and EV2 were immunoblotted for phosphorylated and total content of SMAD3, SMAD1/5/8, Akt, p38 MAPK, ERK1/2, total SMAD4, and total NOX4. GAPDH content was used as a loading control and normalization standard. Note that the cropped version of many of these images is found in Fig 5A and B.

C Phosphorylation of TAK1 is not different between control and AAV8.GDF11-treated hearts or quadriceps.

 © 2017 The Authors

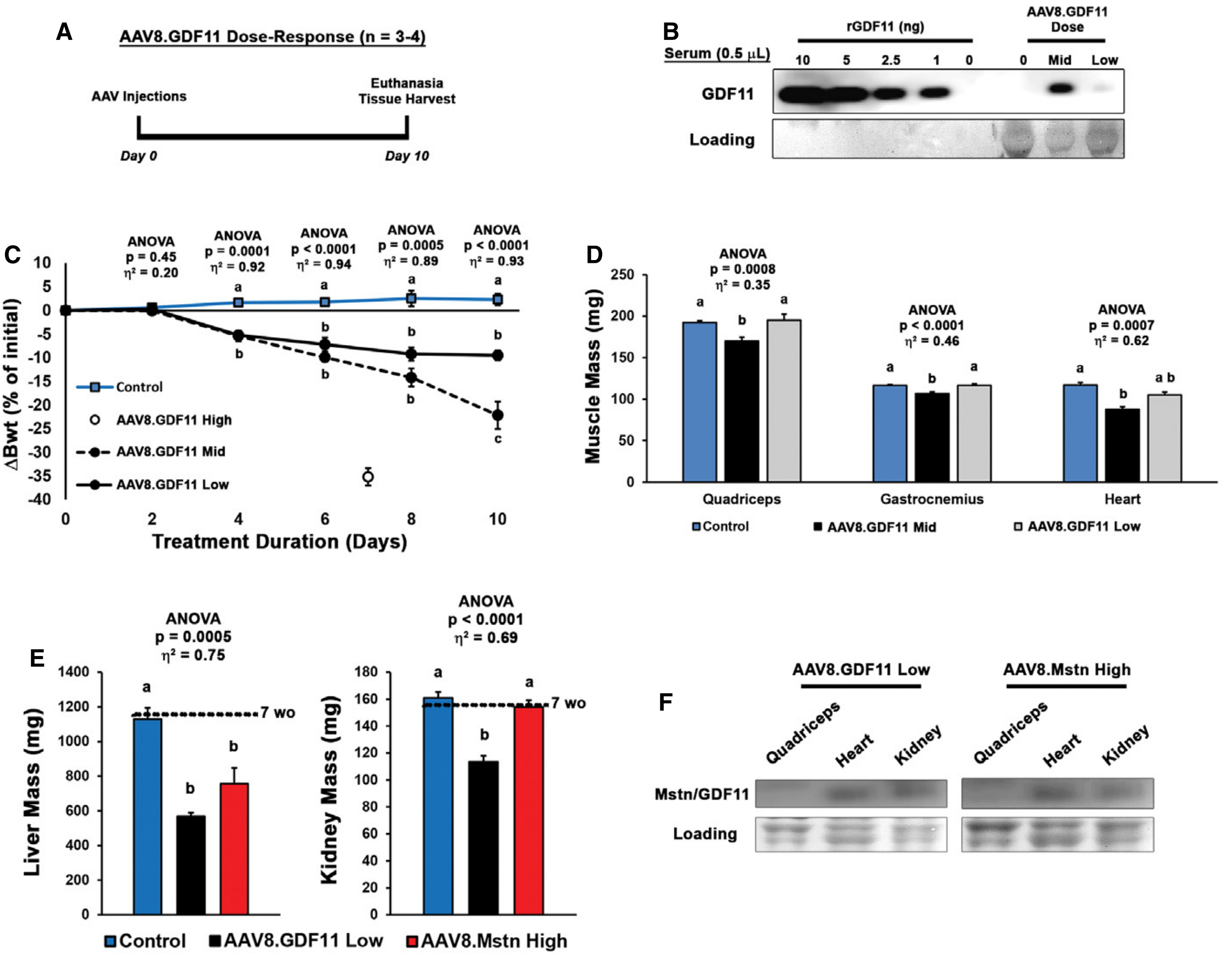

Figure EV5. Lower expression levels of GDF11 induce loss of body weight and muscle atrophy.

A Eleven-week-old C57BL/6 male mice received i.p. injections of PBS (control; n = 4), 1×10^{11} gc of AAV8.GDF11 (mid dose; n = 3), or 5×10^{10} gc of AAV8.GDF11 (low dose; n = 3), and their body weights were monitored every other day until the 1×10^{11} gc AAV8.GDF11 group required euthanasia on day 10.

B Immunoblotting of serum samples for GDF11 demonstrates expression levels of 0.5–1.6 ng/μl and 0.26–0.43 ng/μl for the mid- and low-dose groups, respectively.

C Change in mouse body weights (Bwt) across the 10-day study by the AAV8.GDF11 treatment groups, including terminal values for the previous 7-day cohort treated with 1×10^{12} gc of AAV8.GDF11 (high dose).

D Mass of the quadriceps, gastrocnemius, and heart of the 10-day study mice (n = 12 for control mice by the inclusion of untreated age-matched mice, resulting in a larger, homogenous data set).

E Liver and kidney mass of 7-week-old C57BL/6 male mice were treated with PBS (control; n = 5), AAV8.GDF11 low dose (n = 4), or AAV8.Mstn high dose (n = 5) for 16 days (see Fig 6B). The mean values for 7-week-old mice from this colony (n = 5) are indicated by the dotted line to show starting masses.

F Immunoblotting comparison of monomeric GDF11 and Mstn levels in quadriceps, heart, and kidney demonstrate that differential effects are not due to differential accumulation of the ligands in tissue.

Data information: Values are displayed as mean ± SEM. Statistical analysis performed using one-way ANOVA analysis with Tukey's HSD *post hoc* test (non-connecting letters indicate $P < 0.05$ between groups) and effect size presented as eta-squared (η^2). Note that "b" on Day 4 of panel (C) refers to the 2 overlapping groups.

© 2017 The Authors
